# Liver Cancer 2.0

**DOI:** 10.3390/ijms242417275

**Published:** 2023-12-08

**Authors:** Hiroaki Taniguchi

**Affiliations:** Department of Experimental Embryology, Institute of Genetics and Animal Biotechnology, Polish Academy of Sciences, 05-552 Jastrzebiec, Poland; h.taniguchi@igbzpan.pl

Liver cancer, specifically hepatocellular carcinoma (HCC), is a major global health concern due to its high prevalence in many countries [1]. It is frequently linked to risk factors such as viral infections (hepatitis B and C) [2], alcohol consumption [3], smoking [4], and non-alcoholic fatty liver disease [5]. Notably, studies have demonstrated that aflatoxin exposure leads to liver cancer by forming adducts in DNA sequences and inducing mutations [6,7]. Genetic factors also play a role, and early detection and prevention are crucial because of HCC’s frequent diagnosis at an advanced stage. This Special Issue, entitled “Liver Cancer 2.0”, presents eight papers that offer significant insights into liver cancer, with a particular focus on HCC. These studies provide significant insight into potential therapeutic approaches, shedding light on various aspects of the disease.

In the study by Kim et al., the authors identified LAT1 as a crucial amino acid transporter supporting HCC growth. They used CRISPR/Cas9 to knockout LAT1 in HCC cells, resulting in a significant reduction in cell proliferation and tumor growth. This effect was linked to the inhibition of the mTORC1 signaling pathway. These findings suggest that LAT1 could be a potential therapeutic target for HCC treatment. Another study by Xie et al. explored the role of FAM50A, a DNA-binding protein and transcription factor, in HCC. They found that FAM50A promotes HCC progression and has diagnostic value. Additionally, FAM50A influences the tumor immune microenvironment and immunotherapy outcomes in HCC. The study concludes that FAM50A is an important proto-oncogene in HCC, serving as a diagnostic marker, immunomodulator, and potential therapeutic target. Yuan et al. investigated the impact of genetic variants (single-nucleotide polymorphisms, SNPs) in the long non-coding RNA LINC00673 on HCC risk. They found that in elderly patients (≥60 years), the presence of the LINC00673 rs9914618 A allele was associated with an increased risk of HCC development, and the GA + AA genotype of rs9914618 in elderly patients exhibited a higher risk of having lymph node metastasis. Clinical data also revealed that LINC00673 expression was elevated in HCC tissues, correlated with advanced disease stages and poorer prognoses. The study suggests that the LINC00673 rs9914618 polymorphism may serve as a promising HCC biomarker, particularly in the elderly population. Kanzaki et al. determined the role of the ELAVL1 protein in hepatitis B virus (HBV) infection and HCC. They found that ELAVL1 interacts with HBV-derived RNAs, stabilizing viral RNA and promoting the production of viral proteins. Moreover, ELAVL1 was shown to influence cell growth in HCC cells beyond its role in HBV replication. Clinical analyses of HCC surgical samples indicated that high ELAVL1 expression was associated with HBV-related HCC recurrence. This suggests that ELAVL1 may be a potential therapeutic target for HBV-related HCC treatment.

In addition to the significant advancements in our understanding of the molecular mechanisms and potential therapeutic targets for HCC, this Special Issue also features two therapeutic studies aimed at improving the diagnosis and treatment of liver cancers. Ainora et al. aimed to differentiate between HCC and intracellular cholangiocarcinoma (ICC) using multiparametric ultrasound (MP-US). The study found that MP-US, combining B-Mode ultrasound, dynamic contrast-enhanced ultrasound (D-CEUS), and shear wave elastography (SWE), could accurately distinguish between these two types of liver tumors. D-CEUS parameters, specifically peak intensity (PE), were the most significant in diagnosing HCC. An ultrasound score was developed, offering a non-invasive method for differentiating between HCC and ICC, potentially reducing the need for liver biopsy in some cases. Natural compounds hold promise as potential treatments for liver cancer, offering therapies derived from natural sources with fewer side effects and lower toxicity than conventional chemotherapy. In Wang’s study, researchers focused on HCC and developed a compound called ZS17, a derivative of matrine, which demonstrated strong antiproliferative activity against HCC cells. ZS17 induced apoptosis and increased reactive oxygen species (ROS) production. It activated the ROS-JNK-P53 pathway and the caspase signaling pathway. Both in vitro and in vivo experiments showed promising potential for ZS17 as a chemotherapeutic agent for treating HCC patients, inhibiting tumor growth without significant side effects.

Excellent review and perspective papers are also published in this Special Issue. A review by Zajkowska and Mroczko summarized the involvement of chemokines, particularly CC and CXC chemokines, in the development of HCC. This review highlighted the potential role of chemokines in developing HCC. A perspective article written by Brandi and Rizzo explored the challenges in treating biliary tract cancer and the emerging importance of understanding the tumor microenvironment (TME). The article also discussed potential interactions between IDH inhibitors, immunotherapy, and the TME, focusing on guiding future clinical trial designs.

In summary, the eight papers presented in this Special Issue provide significant insights into liver cancer, with a strong focus on HCC. They cover a wide range of topics, including potential therapeutic targets, genetic factors, diagnostic markers, diagnostic methods, natural compounds for treatment, and the role of chemokines and the tumor microenvironment. These findings contribute to a deeper understanding of liver-related diseases, offering valuable insights for researchers and clinicians in the field and potential avenues for improved diagnosis and treatment.

## Data Availability

Not applicable.
